# Dietary Inflammatory Index and cardiometabolic and inflammatory profiles in children and adolescents with type 1 diabetes: a cross-sectional study

**DOI:** 10.1038/s41390-026-05069-2

**Published:** 2026-05-08

**Authors:** İsmail Mücahit Alptekin, Tuba Ekinci Gezmiş, Fatma Betül Özgeriş, Zehra Şahin, Nurinnisa Öztürk, Zerrin Orbak

**Affiliations:** 1 Department of Nutrition and Dietetics, Faculty of Health Sciences, Atatürk University, Erzurum, Türkiye; 2 Institute of Health Sciences, Atatürk University, Erzurum, Türkiye; 3 Department of Medical Biochemistry, Faculty of Medicine, Atatürk University, Erzurum, Türkiye; 4 Department of Pediatric Endocrinology, Faculty of Medicine, Atatürk University, Erzurum, Türkiye

## Abstract

**Background:**

Nutritional management is central to pediatric type 1 diabetes (T1DM). The Dietary Inflammatory Index (DII) quantifies the inflammatory potential of diet, but evidence in pediatric T1DM remains limited. This study evaluated whether higher DII scores are associated with poorer glycemic control and adverse inflammatory profiles in children and adolescents with T1DM.

**Methods:**

Seventy-eight pediatric patients with T1DM (5–19 years; 59.0% girls) were evaluated using 3-day dietary records, anthropometry, and biomarkers including HbA1c, lipid profile, and inflammatory/adipokine markers (CRP, IL-6, TNF-α, leptin, adiponectin). DII scores were calculated from dietary intake data. Associations between DII and outcomes were tested using covariate-adjusted general linear models controlling for age, sex, BMI, energy intake, and diabetes duration.

**Results:**

In adjusted models, higher DII was independently associated with higher HbA1c (B = 0.48; 95% CI 0.32–0.63; *p* < 0.001), LDL-C (B = 7.62; 95% CI 5.36–9.87; *p* < 0.001), TNF-α (B = 150.58; 95% CI 127.99–173.17; *p* < 0.001), and leptin (B = 730.60; 95% CI 629.14–832.06; *p* < 0.001), and with lower adiponectin (B = − 70.15; 95% CI − 79.93 to −60.37; *p* < 0.001). Higher DII showed greater energy, total fat, saturated fat, and cholesterol, and lower fiber, vitamin A, omega-3, vitamin E, and β-carotene.

**Conclusions:**

More pro-inflammatory dietary profiles were associated with worse metabolic control and higher inflammatory burden, supporting DII as a clinically relevant dimension in pediatric T1DM nutritional assessment.

**Impact:**

- In children/adolescents with T1DM, higher DII is linked to poorer metabolic status, worse glycemic control, and higher BMI/body-composition risk markers.
- In covariate-adjusted models, higher DII is independently associated with higher HbA1c and LDL-C and with an unfavorable adipokine/inflammatory profile (higher TNF-α and leptin, lower adiponectin).
- These associations persist after adjusting for age, sex, BMI, energy intake, and diabetes duration, indicating an independent association with cardiometabolic/inflammatory burden.
- Nutrition care may incorporate dietary inflammatory potential alongside carbohydrate counting; more fiber, unsaturated fats/omega-3, antioxidants; less saturated/ultra-processed foods may be relevant to prospective/interventional testing, potentially to improve long-term pediatric outcomes.

## Introduction

Type 1 diabetes mellitus (T1DM) is a chronic autoimmune disease caused by destruction of pancreatic β-cells, leading to insulin deficiency.^1^ It is common in childhood/adolescence and requires lifelong insulin therapy, blood glucose monitoring, and dietary regulation.^2^ The incidence and prevalence of T1DM in youth have been rising globally; over 1.2 million under 20 years old live with T1DM, and the total number is projected to reach up to 17.4 million by 2040.^3^ In Türkiye, recent data show an incidence of 13.1 per 100,000 children, increasing ~8% annually.^4^

Optimal T1DM management focuses not only on insulin administration but also on nutrition and lifestyle. In addition to carbohydrate counting, the overall quality of the diet has been given attention for its role in systemic inflammation and metabolic health. Accordingly, dietary exposures that modulate inflammatory pathways may be linked to clinically meaningful outcomes in pediatric T1DM, including HbA1c (glycemic control), adiposity (e.g., BMI), lipid profile (e.g., LDL-C), and inflammatory biomarkers.^5^ Chronic low-grade inflammation is implicated in the pathophysiology of various metabolic disorders and diabetes complications,^6^ suggesting that dietary factors that modulate inflammation could impact T1DM outcomes.^7,8^ Diet quality in youth with T1DM has been directly linked to glycemic control and cardiometabolic risk profiles,^9^ supporting the clinical relevance of overall dietary patterns beyond carbohydrate counting.

The Dietary Inflammatory Index (DII) has emerged as a useful tool for evaluating the inflammatory potential of an individual’s diet.^10^ The DII, originally developed by Shivappa et al., is based on a comprehensive review of ~1943 scientific articles linking specific foods and nutrients to inflammatory biomarkers.^11,12^ This index provides a quantitative measure of dietary patterns that can be used to investigate links between diet, inflammation, and health outcomes. In adult populations, higher DII scores have been associated with type 2 diabetes risk, insulin resistance, dyslipidemia, and elevated systemic inflammation, supporting its relevance to metabolic health and inflammatory burden.^13–15^

Applying the DII in pediatric T1DM is important, as diet is key in management, and inflammation may affect metabolic control. Evidence shows that a more pro-inflammatory diet (higher DII scores) in children and adolescents with T1DM is linked to higher BMI, elevated lipid levels, and a trend toward poorer HbA1c.^8^ These findings suggest that an anti-inflammatory diet may support better weight, lipid profile, and possibly blood glucose control.

Nevertheless, the pediatric T1DM literature remains limited in two key respects. First, relatively few studies have evaluated DII in relation to both glycemic control and a broad inflammatory/adipokine panel in the same cohort. Second, many existing reports provide limited multivariable adjustment, leaving uncertainty about whether observed associations persist after accounting for clinically relevant factors (e.g., age, sex, anthropometry, energy intake, and diabetes-related characteristics). Therefore, the present study aimed to assess the association between DII (applied to pediatric dietary intake) and HbA1c, lipid profile, anthropometric indices, and inflammatory/adipokine biomarkers (CRP, IL-6, TNF-α, leptin, adiponectin) in children and adolescents with T1DM. We hypothesized that a higher DII score would be associated with higher HbA1c, higher LDL-C, higher BMI/adiposity, and higher pro-inflammatory biomarkers, alongside a less favorable adipokine profile (higher leptin and lower adiponectin).

## Methods

### Design and study sample

This cross-sectional study was conducted with volunteer children and adolescents aged 5–19 years who were diagnosed with T1DM and followed up at the Faculty of Medicine, Pediatric Endocrinology Clinic of Atatürk University Research Hospital between 01 June 2024 and 30 March 2025. Exclusion criteria for the study were: current use of non-steroidal anti-inflammatory drugs, statins, or antihypertensive medications; use of any vitamin and/or mineral supplements; having a severe psychiatric illness or use of psychiatric medications; presence of endocrine, kidney, or liver diseases; having an acute febrile illness; a diagnosis of inflammatory bowel disease, rheumatoid arthritis, or cancer; a diagnosis of any other autoimmune disease.

The sample size was calculated using the G*Power (v3.1.9.7). Based on a previous study that examined the effects of adherence to a Mediterranean diet (known to have anti-inflammatory effects) on metabolic parameters in individuals with T1DM, a difference of 0.5% in HbA1c (with standard deviations of 0.55 and 0.97) was used for the power analysis. With a 5% significance level and 80% power, the required sample size was estimated to be 82 participants.^9^ The current study was completed with 78 participants. Ethical approval was obtained from the Atatürk University Non-Interventional Clinical Research Ethics Committee (Approval Number: B.30.2.ATA.0.01.00/277, Date: 03.05.2024). Informed consent was obtained from all patients and parents included in this study. Participants were provided with detailed information regarding the study’s aim, procedures, potential risks, and confidentiality measures before giving their consent. This study was financially supported by The Scientific and Technological Research Council of Türkiye (TÜBİTAK) with project number 124S341.

**Fig. 1:**
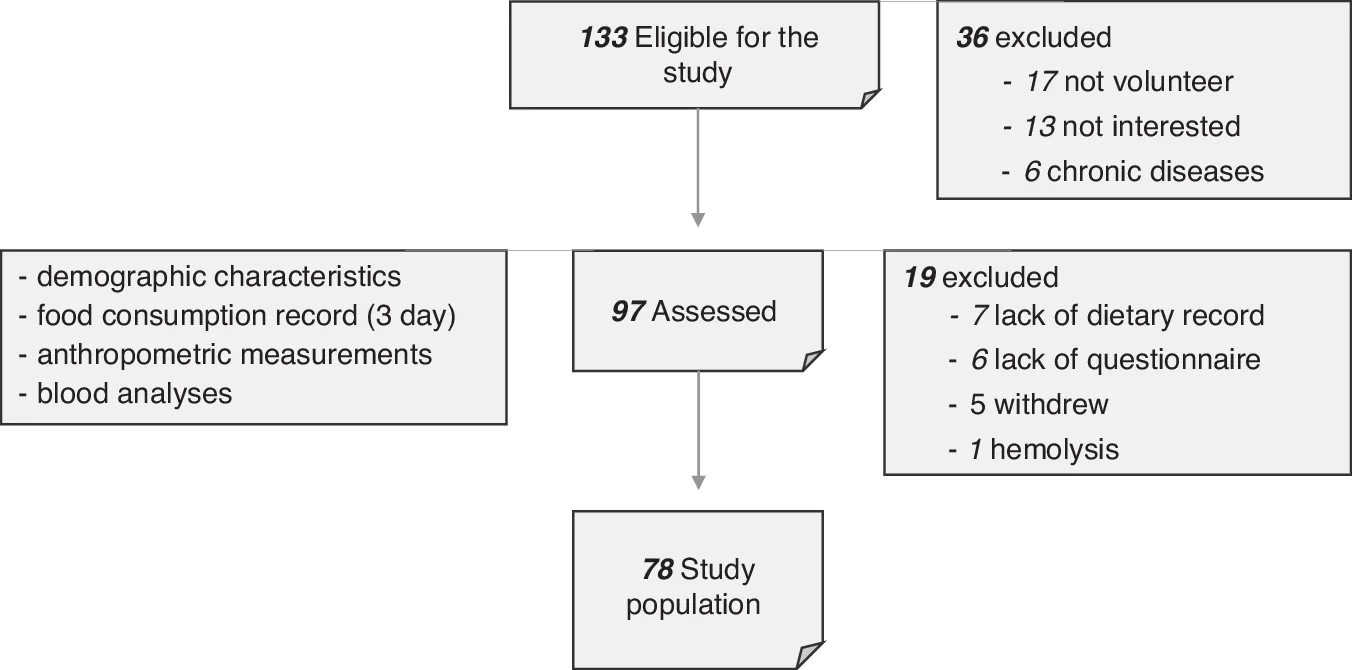
Flow diagram for the study participants.

The flow diagram for the study participants is shown in Fig. 1. The general plan of the study was as follows: Descriptive information about the participants, family-related information, dietary habits, and food consumption records were collected using a structured questionnaire administered face-to-face to the participants and their parents. The researchers also measured anthropometrics, including body weight, height, waist circumference, hip circumference, and mid-upper arm circumference for each participant. After the demographic data, dietary habits, anthropometric measurements, and food intake records were obtained, fasting blood samples were collected from each participant as the final step of data collection. While the vast majority of individuals received carbohydrate counting training, insulin use varied depending on carbohydrate counting and insulin sensitivity factors; therefore, data on this could not be obtained from individuals and thus could not be included in the findings and analyses.

### Measurements

#### Demographics

In the first part of the questionnaire, demographic (age, sex, age at diagnosis, year of diagnosis, and knowledge/application of carbohydrate counting) and family information (parental age, occupation, education level, socioeconomic status, and number of family members) were collected.

#### Dietary assessment

The second part of the questionnaire addressed the dietary habits of the participants. Questions were asked about whether the participant followed any specific diet, the number of main meals and snacks consumed per day, whether any meals were skipped (and the reasons for skipping), the types of foods preferred for snacks, and the frequency of consumption of various foods. In addition to accurately assessing dietary intake and calculating the DII, each participant’s food intake was recorded using a 3-day food diary (two weekdays and one weekend). At the beginning of the study, trained and registered dietitians guided parents in completing 24-h dietary recalls using food models and portion-size visuals. During the recall periods, we collected three 24-h dietary recall data. Dietary intake data were analyzed using the Beslenme Bilgi Sistemi (BeBiS) version 9.0.

#### Anthropometric measurements

The anthropometric measurements of participants included body weight (kg), height (cm), BMI (kg/m^2^), waist circumference (cm), waist-to-height ratio, hip circumference (cm), skinfold thicknesses (triceps and biceps, in cm), mid-upper arm circumference (MUAC, cm), body fat percentage (%), fat mass (kg), fat-free (lean) body mass (kg), muscle mass (kg), total body water (kg), and total body water percentage (%). All anthropometrics were measured by the researchers at the hospital.

Body weight, body fat percentage, fat mass, fat-free mass, muscle mass, total body water, and total body water percentage were measured in the morning with the participant fasting, wearing light clothing, and without shoes or socks. A Tanita MC-780MA segmental body composition analyzer was used, which operates on the bioelectrical impedance analysis (BIA) principle. Participants were instructed to avoid heavy exercise for 24–48 h before the measurement, to refrain from excessive fluid intake in the 8–12 h before measurement, and to come in a fasting state on the measurement day.

Height was measured without shoes, with the participant standing upright in the Frankfort horizontal plane (the line from the lower border of the eye orbit to the upper border of the ear canal is horizontal) using a mechanical stadiometer. BMI was calculated as weight (kg/m2) and categorized according to WHO BMI-for-age z-scores: underweight (<−2 SD), normal weight (−2 to +1 SD), and overweight/obese (>+1 SD).^16^ These calculations were performed using the WHO AnthroPlus program.

Waist circumference was measured at the midpoint between the lowest rib and the iliac crest using a non-elastic measuring tape while the participant was standing. The waist-to-height ratio was calculated as waist circumference divided by height; a ratio of <0.5 was considered “normal” and a ratio ≥0.5 was considered an indication for intervention.^17^ Hip circumference was measured with the child standing, by wrapping a measuring tape around the widest part of the hips/buttocks, ensuring the tape was horizontal. Skinfold thicknesses at the triceps and biceps were measured using a Holtain caliper (Holtain Ltd, UK) with standard procedures.^18^

#### Dietary inflammatory index (DII)

The Dietary Inflammatory Index (DII) was calculated to assess the inflammatory potential of each participant’s diet. For this calculation, the method described by Shivappa et al. was used.^11^ In brief, this method requires a “standard global mean” and standard deviation for each of a set of food parameters/nutrients, which were obtained by Shivappa and colleagues from extensive datasets of dietary intake from different populations around the world. In our study, the DII was computed based on the intake of various nutrients obtained from the 3-day dietary records. A total of 45 dietary parameters are considered in the full DII; in this study, the nutrients available from the records and used for DII calculation included energy, protein, total fat, saturated fatty acids, monounsaturated fatty acids, polyunsaturated fatty acids, omega-3 fatty acids, omega-6 fatty acids, cholesterol, carbohydrate, total dietary fiber, vitamin A, beta-carotene, vitamin E, thiamine (vitamin B1), riboflavin (vitamin B2), niacin (vitamin B3), pyridoxine (vitamin B6), cobalamin (vitamin B12), vitamin C, folic acid, vitamin D, iron, zinc, magnesium, and selenium.^10^ For each participant, the intake amount of each nutrient was first standardized into a z-score: this was done by subtracting the global mean intake of that nutrient from the participant’s intake and dividing by the global standard deviation (Supplementary Material, Table S1). These z-scores were then converted to percentile scores. Each percentile score was centered (zero-based) and multiplied by the corresponding “overall inflammatory effect score” for that nutrient. Finally, the scores for all nutrients were summed to obtain the individual’s overall DII score, which reflects the inflammatory potential of the diet.^19^ Participants were then divided into tertiles (T1, T2, T3) based on their DII scores, and the group with the lowest DII score (most anti-inflammatory diet) was considered the reference (first tertile) to which the other groups were compared.^20^

#### Blood analyses

Fasting blood samples (10 mL) were collected from participants in gel separator tubes, allowed to clot for 30–45 minutes, and centrifuged at 3000 rpm for 10 min. Serum was separated, aliquoted in triplicate, and stored at –80 °C until analysis. Biochemical measurements, including fasting glucose, HDL, LDL, triglycerides, HbA1c, CRP, and fibrinogen, were performed using a fully automated biochemical analyzer. Additional analyses measured serum adiponectin (Shanghai Korain Biotech Co., Ltd., Cat. No: E1550Hu), TNF-α (Cat. No: E0082Hu), IL-6 (Cat. No: E0090Hu), and Leptin (Cat. No: E1559Hu) via commercial Sandwich ELISA kits (Shanghai Korain Biotech Co. Ltd.), in duplicate, following manufacturers’ instructions using a Multiskan SkyHigh Microplate Spectrophotometer (Thermo Fisher Scientific).

### Statistical analysis

Statistical analyses were performed using the Statistical Package for Social Sciences (SPSS) software version 26 (IBM SPSS Inc., Chicago, IL). A *p*-value of <0.05 was considered statistically significant. The distribution of continuous variables was evaluated using the Kolmogorov-Smirnov test to assess normality. For variables that showed an approximate normal distribution, results were expressed as mean and standard deviation. Categorical variables were expressed as a number (*n*) and percentage (%).

Participants were categorized into tertiles of DII scores (T1: low, T2: moderate, T3: high). Between-tertile differences in continuous variables were evaluated using one-way analysis of variance (ANOVA) with Bonferroni post-hoc tests, and categorical variables were compared using the chi-square test. Given the strong potential for confounding by age, sex, BMI, diabetes duration, and energy intake in pediatric populations, tertile-based comparisons were considered descriptive. Primary inference relied on prespecified covariate-adjusted models with DII as the main exposure. For each biochemical and inflammatory marker, univariate general linear models (GLM) were fitted, including DII and the following covariates: age, sex, BMI, energy intake, and year of diagnosis. Unstandardized regression coefficients (B), 95% confidence intervals (CI), and p values were reported. Correlation analyses were performed only for exploratory/descriptive purposes and are provided in the Supplementary material (Table S2).

## Results

### Participant characteristics

Participant characteristics are summarized in Table 1. Among 78 participants, 59.0% were girls (*n* = 46). Sex distribution, diabetes history (including time since diagnosis), and carbohydrate counting training did not differ across DII tertiles (all *p* > 0.05). Socioeconomic indicators and parental characteristics (income, parental education and employment, household size) were also comparable between tertiles (all *p* > 0.05). Age differed across tertiles (*p* = 0.035), with participants in T2 being younger on average than those in T3; therefore, subsequent comparisons were interpreted with attention to age-related differences in growth and energy requirements.

**Table 1:** Participant characteristics according to the dietary inflammatory index tertiles.

*n* (%)	T1	T2	T3	*p* value
Sex				*χ*²= 5.511 *p* = 0.064
Girl	12 (26.1)	14 (30.4)	20 (43.5)	
Boy	14 (43.8)	12 (37.5)	6 (18.8)	
Carbohydrate counting training				*χ*²= 1.884 *p* = 0.390
Yes	15 (28.3)	19 (35.8)	19 (35.8)	
No	11 (44.0)	7 (28.0)	7 (28.0)	
Income level				*χ*²= 6.500 *p* = 0.165
Income is less than expenses	19 (39.6)	17 (35.3)	12 (25.0)	
Income is equal to expenses	4 (25.0)	3 (18.8)	9 (56.3)	
Income is greater than expenses	3 (21.4)	6 (42.9)	5 (35.7)	
Mother education				*χ*²= 6.592 *p* = 0.159
Primary school	22 (40.0)	15 (27.3)	18 (32.7)	
High school	1 (7.7)	6 (46.2)	6 (46.2)	
University	3 (30.0)	5 (50.0)	2 (20.0)	
Father education				*χ*²= 2.281 *p* = 0.588
Primary school	13 (33.3)	12 (30.8)	14 (35.9)	
High school	11 (40.7)	8 (29.6)	8 (29.6)	
University	2 (16.7)	6 (50.0)	4 (33.3)	
Mother’s employment status				*χ*²= 14.03 *p* = 0.171
Yes	7 (36.8)	6 (31.6)	6 (31.6)	
No	19 (32.2)	20 (33.9)	20 (33.9)	
Father’s employment status				*χ*²= 6.542 *p* = 0.587
Yes	25 (33.3)	24 (32)	26 (34.6)	
No	1 (33.3)	2 (66.7)	-	
**Mean (SD)**				
Age (years)	12.4 (2.62) ^a, b^	10.7 (3.29) ^a^	13.0 (3.46) ^b^	**0.035**
Year of diagnosis (years)	3.46 (3.50)	3.31 (2.89)	3.31 (2.79)	0.979
Mother age (years)	41.4 (6.73)	37.3 (6.27)	40.8 (4.70)	0.054
Father age (years)	45.8 (6.57) ^a, b^	42.2 (6.57) ^a^	47.1 (6.54) ^b^	**0.027**
Number of individuals in the family	5.35 (1.62)	5.27 (1.80)	5.15 (1.34)	0.910
Number of children in the family	3.77 (1.50)	3.23 (1.47)	2.88 (1.14)	0.074
Ranking of the patient in the family	2.92 (1.76) ^a^	2.31 (1.59) ^a, b^	2.38 (1.48) ^b^	**0.048**

T1 (−1.55; 1.00), T2 (1.04; 2.03), and T3 (2.04; 2.80) are tertiles. Statistically significant results are indicated in bold letters.

### Anthropometric measurements

Anthropometric and body composition measures by DII tertiles are presented in Table 2. BMI differed significantly across tertiles (*p* < 0.001), with the highest BMI observed in the highest DII tertile (T3). The waist-to-height ratio also differed across tertiles (*p* = 0.020). Differences observed in absolute body weight across tertiles were not emphasized because weight is strongly age-dependent and tertiles differed by age; thus, BMI (and related indices) and adjusted models were prioritized for interpretation. Several body composition measures (fat-free mass, muscle mass, and total body water) showed statistically significant differences across tertiles (all *p* < 0.05), whereas other anthropometric measures were broadly similar across groups (Table 2).

**Table 2:** Anthropometric and body composition characteristics of participants across dietary inflammatory index tertiles.

*n* (%)	T1	T2	T3	*p* value
Weight (kg)	41.2 (12.9) ^a,b^	38.1 (13.9) ^b^	48.4 (17.7) ^a^	**0.047**
Height (cm)	152.1 (14.2)	142.6 (16.4)	151.3 (15.7)	0.056
Body mass index (kg/m^2^)	14.9 (1.42) ^a^	18.3 (2.17) ^b^	22.8 (4.08) ^c^	**<0.001**
Waist circumference (cm)	65.8 (7.37)	67.4 (11.1)	70.5 (12.5)	0.265
Waist-to-height ratio	0.433 (0.03) ^a^	0.473 (0.05) ^b^	0.466 (0.06) ^a,b^	**0.020**
Hip circumference (cm)	79.5 (11.7)	79.2 (12.4)	86.1 (14.7)	0.110
Triceps skinfold thicknesses (cm)	11.9 (3.70)	12.6 (4.44)	14.5 (5.11)	0.096
Biceps skinfold thicknesses (cm)	12.8 (5.74)	15.2 (5.76)	14.6 (5.11)	0.284
Mid-upper arm circumference (cm)	21.6 (2.90)	22.0 (4.39)	24.5 (5.33)	0.056
Body fat percentage (%)	6.43 (4.87)	7.96 (5.36)	10.6 (9.11)	0.081
Fat-free body mass (kg)	35.1 (9.84) ^a,b^	30.2 (9.39) ^b^	37.7 (11.4) ^a^	**0.032**
Muscle mass (kg)	33.3 (9.27) ^a,b^	28.5 (8.93) ^b^	35.8 (10.1) ^a^	**0.031**
Total body water (kg)	25.6 (7.20) ^a,b^	22.1(6.80) ^b^	27.5 (8.49) ^a^	**0.036**

T1 (-1.55; 1.00), T2 (1.04; 2.03), and T3 (2.04; 2.80) are tertiles. Statistically significant results are indicated in bold letters.

### Nutrient intake patterns

Energy and nutrient intakes across tertiles are shown in Table 3. Participants with higher DII scores had diets characterized by higher total energy intake and higher intakes of macronutrients (notably total fat and cholesterol) (*p* < 0.05 for key contrasts), alongside lower fiber and lower intakes of several micronutrients with antioxidant/anti-inflammatory relevance (including vitamins A, C, E, β-carotene, and vitamin D) (*p* < 0.05). Overall, higher DII tertiles reflected a dietary pattern with greater energy density and a less favorable anti-inflammatory nutrient profile.

**Table 3:** Energy and nutrient intakes across the dietary inflammatory index tertiles.

Mean (standard deviation)	T1	T2	T3	*p* value
Energy (kkal)	1429 (371)	1732 (316)	2073 (372)	**<0.001**
Water (ml)	2176 (679)	1857 (627)	2034 (674)	0.225
Protein (g)	51.3 (13.3)	62.8 (22.2)	73.5 (26.5)	**0.020**
Total fat (g)	65.1 (12.4)	75.1 (6.7)	84.2 (10.1)	**<0.001**
Saturated fat (g)	20.1 (8.60)	25.6 (13.3)	26.3 (9.3)	0.070
Mono unsaturated fat (g)	24.2 (5.78)	25.1 (6.42)	29.3 (6.71)	**0.001**
Polyunsaturated fat (g)	16.5 (5.23)	18.5 (5.20)	23.5 (6.52)	**<0.001**
n-3 (g)	1.60 (0.42)	1.40 (0.56)	0.91 (0.41)	**<0.001**
n-6 (g)	4.18 (2.10)	6.97 (3.60)	6.36 (3.20)	**0.004**
Cholesterol (mg)	172 (34.6)	225 (42.9)	275 (61.1)	**<0.001**
Carbohydrates (g)	181.2 (43.4)	223.6 (36.1)	262.5 (45.2)	**<0.001**
Dietary fiber (g)	18.2 (4.49)	15.7 (3.21)	11.5 (4.64)	**<0.001**
Vitamin A (RE)	923 (211)	765 (186)	611 (190)	**<0.001**
Beta-Karoten (μg)	5.19 (1.61)	(3.29)	2.43 (1.93)	**<0.001**
Vitamin D (μg)	1.19 (0.39)	1.31 (0.58)	0.88 (0.32)	**0.003**
Vitamin E (mg)	20.5 (7.28)	15.5 (6.95)	10.4 (4.50)	**<0.001**
Thiamin (mg)	0.85 (0.44)	0.75 (0.31)	0.74 (0.51)	0.610
Riboflavin (mg)	1.46 (0.38)	1.32 (0.37)	1.02 (0,45)	**0.001**
Niacin (mg)	11.7 (3.11)	9.99 (3.40)	7.21 (2.36)	**<0.001**
Vitamin B6 (mg)	1.27 (0.33)	1.04 (0.36)	0.81 (0.31)	**<0.001**
Folic acid (μg)	156 (53.8)	149 (40.8)	110 (37.6)	**0.001**
Vitamin B12 (μg)	2.24 (1.08)	3.57 (1.37)	4.01 (1.37)	**<0.001**
Vitamin C (mg)	148 (39.2)	104 (42.5)	92.5 (59.7)	**<0.001**
Iron (mg)	7.35 (2.17)	8.51 (2.51)	10.8 (3.52)	**<0.001**
Magnesium (mg)	228 (57.4)	216 (59.1)	200 (61.8)	**<0.001**
Zinc (mg)	8.00 (2.59)	6.46 (2.50)	7.91 (2.68)	**<0.001**
Selenium (mg)	0.232 (0.047)	0.153 (0.018)	0.115 (0.023)	**<0.001**

T1 (-1.55; 1.00), T2 (1.04; 2.03), and T3 (2.04; 2.80) are tertiles. Statistically significant results are indicated in bold letters.

### Biochemical and inflammatory markers

Unadjusted comparisons of cardiometabolic and inflammatory markers across DII tertiles are provided for descriptive purposes (Supplementary Material, Table S3). Higher DII tertiles were associated with markedly worse glycemic and lipid-related profiles: HbA1c and LDL-C increased across tertiles (*p* < 0.001). In contrast, HDL-C, triglycerides, total cholesterol, fasting glucose, CRP, and fibrinogen did not differ significantly across tertiles (all *p* > 0.05). For inflammatory/adipokine markers, higher DII tertiles were associated with higher TNF-α, higher IL-6, and higher leptin, and lower adiponectin (all *p* < 0.001), consistent with a more pro-inflammatory profile.

### Adjusted associations between DII and outcomes

To address confounding, we additionally performed adjusted models with DII as the primary exposure and age, sex, BMI, energy intake, and diagnosis timing as covariates (Table 4). In these adjusted analyses, higher DII was independently associated with higher HbA1c (*B* = 0.48, 95% CI 0.32–0.63; *p* < 0.001) and LDL-C (*B* = 7.62, 95% CI 5.36–9.87; *p* < 0.001), as well as higher TNF-α (B = 150.58, 95% CI 127.99–173.17; *p* < 0.001) and leptin (*B* = 730.60, 95% CI 629.14–832.06; *p* < 0.001). Conversely, DII was inversely associated with adiponectin (*B* = − 70.15, 95% CI − 79.93 to −60.37; *p* < 0.001). No independent associations were observed between DII and HDL-C, triglycerides, total cholesterol, CRP, fibrinogen, or IL-6 after adjustment (all *p* > 0.05).

**Table 4:** Adjusted association between DII and biochemical and inflammatory markers.

Outcome	B (DII)	95% CI	*p* value
HbA1c (%)	0.47	0.32 to 0.63	**<0.001**
HDL (mg/dL)	1.58	−3.80 to 6.95	0.560
LDL (mg/dL)	7.62	5.36 to 9.87	**<0.001**
TG (mg/dL)	6.72	−11.33 to 24.76	0.460
Total cholesterol (mg/dL)	2.90	−5.76 to 11.56	0.506
CRP (mg/dL)	0.46	−0.66 to 1.59	0.416
Fibrinogen (mg/dL)	−5.33	−15.88 to 5.22	0.317
Adiponectin (µg/mL)	−70.15	−79.93 to −60.37	**<0.001**
TNF-α (pg/mL)	150.58	127.99 to 173.17	**<0.001**
IL-6 (pg/ml)	−0.25	−0.84 to 0.33	0.395
Leptin (ng/mL)	730.60	629.14 to 832.06	**<0.001**

Models adjusted for age, sex, BMI, energy intake, and diabetes duration. Statistically significant results are indicated in bold letters.

## Discussion

In this pediatric T1DM cohort, a more pro-inflammatory diet, as determined by DII, was associated with worse glycemic control, greater adiposity, higher LDL-C, and higher inflammatory activity (TNF-α). In covariate-adjusted models, higher DII was independently associated with higher HbA1c, LDL-C, TNF-α, and leptin, and with lower adiponectin, whereas associations with HDL-C, triglycerides, total cholesterol, CRP, fibrinogen, and IL-6 were not significant. Nevertheless, because of the cross-sectional design, these results should be interpreted as associations rather than causal effects.

### Dietary patterns and quality

Participants with higher DII consumed more total energy, saturated fat, cholesterol, and refined carbohydrates, and less fiber, antioxidant vitamins (A, C, E, beta-carotene), and anti-inflammatory minerals (magnesium, zinc, selenium). This nutrient profile is characteristic of Western diets, known for pro-inflammatory effects and links with obesity, diabetes, and cardiovascular disease.^21,22^ In contrast, Mediterranean-like patterns provide fiber and unsaturated fats linked with lower inflammation.^23^ Our findings support this. The higher mean DII ( + 1.4) compared with some pediatric cohorts^19,24–26^ suggests suboptimal diet quality in our sample.

It is worth noting that the older adolescents in our sample tended to have higher DII scores than the younger children. Age-related differences (higher DII in older adolescents) likely reflect increased autonomy and ultra-processed food exposure.^27,28^ Our findings align with this behavior pattern, they may adopt more Westernized eating habits that drive up their DII. On the other hand, we did not observe significant differences in DII by sex or associations with socioeconomic indicators like parental education or household income, indicating a cross-cutting issue across demographics.^6,29^ However, an important nuance is that we used the standard DII algorithm, which standardizes intakes to a global reference database largely derived from adult dietary data. Older adolescents (15–19 years) may be reasonably comparable to adults in dietary quantity and composition, making adult reference standardization more appropriate. Younger children (5–14 years) may have systematically lower energy intake than adults; therefore, adult standardization may yield age-related measurement distortion or misclassification. Because of this, interpretation of “higher DII in older adolescents” should be cautious, and we avoided over-interpreting age-based tertile differences. Accordingly, age-related tertile differences were interpreted descriptively rather than as definitive evidence of changes in dietary inflammatory potential. Future studies should consider pediatric-specific inflammatory diet indices (where age-appropriate) and age-stratified analyses to improve comparability across developmental stages.

### DII and adiposity

In descriptive analyses, participants in higher DII categories tended to have higher BMI and less favorable body composition indicators. In adjusted analyses of BMI (adjusted for age, sex, energy intake, and diabetes duration), DII remained independently associated with BMI, suggesting that dietary inflammatory potential may relate to adiposity beyond total energy intake. These findings align with pediatric reports linking pro-inflammatory diets to overweight/obesity.^30,31^ Serum leptin was also elevated in the high-DII group, supporting links between diet-related inflammation and adipokine signaling.^32^ Together, these findings suggest that pro-inflammatory diets may exacerbate adiposity and related endocrine changes in pediatric T1DM, whereas anti-inflammatory, nutrient-dense diets might help protect against excess weight gain.

### Glycemic control

One of the most striking findings was the strong association between DII and HbA1c. Children in the highest DII tertile had average HbA1c levels of ~12.4%, compared to ~7.4% in the lowest tertile—a clinically critical difference.^33^ This association remained in covariate-adjusted analyses (Table 4). Therefore, while the adjusted association strengthens inference, residual confounding by treatment regimen and adherence cannot be excluded. This underscores that higher refined carbohydrates and saturated fat, along with lower fiber/omega-3, may relate to insulin resistance and oxidative stress, whereas fiber and anti-inflammatory nutrients are linked with improved insulin sensitivity.^34–38^ Our findings are in line with emerging evidence that diet quality plays a key role in diabetes management. Evidence from Mediterranean diet studies supports better HbA1c outcomes in T1DM and Type 2 diabetes.^39,40^ While causal inference is not possible, our adjusted findings suggest that the diet’s inflammatory potential may be relevant to glycemic control alongside adiposity. Thus, the pro-inflammatory dietary pattern is likely exacerbating hyperglycemia and insulin resistance in youths, whereas shifting toward an anti-inflammatory eating pattern could substantially improve their metabolic control. These results suggest that, beyond carbohydrate counting, overall dietary quality and inflammatory potential influence glycemic outcomes.

A key concern is that older adolescents generally have greater autonomy, more frequent eating outside the home, and higher exposure to ultra-processed foods. At the same time, adolescence is also a period in which treatment non-adherence and reduced caregiver oversight are common, potentially worsening HbA1c independently of diet.^41^ Because insulin regimen type, insulin dose, and objective adherence measures were not captured, confounding is possible. Therefore, the DII–HbA1c association may partly reflect unmeasured variability in diabetes self-management rather than diet alone. When compared with existing literature, DII–HbA1c evidence appears heterogeneous. While some studies report significant associations between pro-inflammatory diets and poorer glycemic outcomes, other studies—particularly in adult populations—have reported weak or non-significant relationships after adjustment. This variability suggests that diet–HbA1c associations likely depend on some factors, including age, treatment adherence, and metabolic status.^42^

### Lipid profile

LDL-C was higher among participants with more pro-inflammatory dietary profiles, and this pattern remained evident in covariate-adjusted analyses (Table 4), consistent with their higher saturated fat and cholesterol intake. Given the established effects of saturated fat on LDL receptor activity and hepatic LDL production,^43–45^ and the LDL-lowering influence of unsaturated fats and fiber,^46^ our results are consistent with diet-lipid biology and raise concern for early atherogenic risk in youth.^47–49^ However, evidence on DII and lipid fractions is heterogeneous, and some large population-based studies have reported weak or non-significant associations with LDL-C after adjustment.^50^ Interestingly, we did not observe significant differences in HDL cholesterol, triglycerides, or total cholesterol between DII groups. The lack of change in HDL and TG might indicate that those parameters are influenced by factors beyond diet alone (such as genetics, physical activity, etc.).

### Inflammatory biomarkers

A key outcome of our study is that a pro-inflammatory diet was indeed associated with heightened inflammation biomarkers. Inflammatory cytokines (TNF-α, IL-6) were higher with pro-inflammatory diets. The TNF-α and IL-6 concentrations were, on average, 3–5 times higher in the highest DII group compared to the lowest. In covariate-adjusted models, DII showed a strong independent association with higher TNF-α. Although IL-6 differed across tertiles descriptively, the adjusted association between DII and IL-6 was not significant, suggesting that the IL-6 pattern may be partly explained by covariates such as adiposity, age, or diabetes-related factors.^51^ Pro-inflammatory diets were associated with significantly higher IL-6 and TNF-α, two key cytokines driving systemic inflammation, insulin resistance, and endothelial dysfunction.^52,53^ Our findings align with previous pediatric DII studies showing higher inflammatory markers in children with pro-inflammatory diets.^19,54,55^ By contrast, those adhering to healthier (e.g., DASH or Mediterranean) diets show lower levels of these cytokines.^53^ Notably, children who were overweight/obese and consuming high pro-inflammatory diets have been suggested to exhibit high inflammatory cytokine levels.^56^ This suggests an interaction between poor diet and adiposity in driving inflammation, which is a potential mechanism that may also be present in our high DII group, given their higher BMIs.

In adjusted analyses, higher DII was associated with lower adiponectin and higher leptin, supporting a more unfavorable adipokine profile with pro-inflammatory dietary patterns. This combination—low adiponectin and high leptin—is a hallmark of the pro-inflammatory, insulin-resistant state often seen in obesity and metabolic syndrome.^20^ Adiponectin is known for its anti-inflammatory and insulin-sensitizing properties,^57^ it enhances insulin sensitivity, promotes fatty acid oxidation, and exerts anti-diabetic and anti-atherogenic.^28^ High adiponectin levels are associated with better glycemic control and less inflammation, whereas low adiponectin (as seen in our high DII group) can remove this protective effect. Leptin is generally pro-inflammatory; it is often elevated in obesity and can stimulate the production of inflammatory cytokines while also reflecting excess fat mass.^58^ In our T1DM children, those consuming pro-inflammatory diets not only had higher adiposity, but the diet itself may contribute to a state of leptin resistance and leptin overproduction, which has been implicated in chronic inflammation. The adiponectin–leptin axis is thus tilted in an unhealthy direction with a pro-inflammatory diet: adiponectin is suppressed, and leptin is elevated, which is linked to worsened insulin resistance and inflammation. Supporting our findings, a study in overweight/obese adults demonstrated the same pattern – higher DII scores were significantly associated with lower adiponectin and higher leptin, even after adjusting for BMI.^59^ This suggests that diet-driven inflammation can independently affect adipokine secretion and action.

Consistent with the tertile comparisons, CRP and fibrinogen were not independently associated with DII in covariate-adjusted models, which may reflect the limited sensitivity of acute-phase reactants to chronic low-grade dietary inflammation in this age group,^60^ but in the context of chronic low-grade inflammation, their elevations may be more subtle or inconsistent. It’s possible that the chronic inflammatory state induced by diet is related to cytokine levels (IL-6, TNF-α) and adipokines, whereas CRP and fibrinogen might not have been elevated in these young patients unless an acute inflammatory trigger was present. In some studies, dietary indices correlate less strongly with CRP, likely due to CRP’s short-term responsiveness and individual variability.^61^ Shivappa et al. (2014) noted positive associations between DII and CRP in adults,^11^ but in pediatric patients with T1DM, CRP may not always reflect the changes in IL-6/TNF-α. Additionally, our T1DM participants, even with pro-inflammatory diets, might not have had significantly elevated CRP because they were relatively well at the time (no active infections or acute illnesses).

### Clinical implications

These associations support considering dietary inflammatory load—not only macronutrient counting—in pediatric T1DM counseling.^62^ Although our study was cross-sectional, the strong associations we observed suggest that if one were to improve a DII score (i.e., shift their diet to be more anti-inflammatory), one might expect improvements in weight control, HbA1c, LDL cholesterol, and inflammatory marker levels. Strategies (fiber-rich whole foods, unsaturated fats, omega-3 sources; reduced saturated fat, refined carbohydrates, and ultra-processed foods) may help improve glycemic and inflammatory profiles. Any benefits require confirmation in prospective/interventional studies.

### Strengths and limitations

Strengths in this study include evaluation of multiple metabolic and inflammatory outcomes in pediatric T1DM, including cytokines and adipokines, use of 3-day dietary records supported by dietitian instruction, and use of covariate-adjusted general linear models across a broad biomarker panel, adjusting for age, sex, BMI, energy intake, and diabetes duration. However, this study has several limitations. This cross-sectional, single-center study with a modest sample size limits causal inference and generalizability. Three-day dietary records are susceptible to recall/under-reporting and may not capture habitual intake. Residual confounding cannot be excluded. Also, there are important unmeasured confounders such as pubertal stage, insulin regimen characteristics, and treatment adherence. Future longitudinal and intervention studies are needed to test whether altering DII leads to improvements in BMI, HbA1c, LDL-C, and cytokine profiles.

## Conclusion

In summary, higher DII was associated with greater adiposity, higher HbA1c, more atherogenic LDL-C, and elevated TNF-α in pediatric T1DM, with associations persisting in adjusted models. These data highlight the potential clinical relevance of the diet’s inflammatory profile and motivate interventional studies targeting dietary inflammation to support metabolic and inflammatory outcomes.

## Supplementary information

Supplementary information

## Data Availability

All data generated or analyzed during this study are included in this published article.
