# Supplementary material for: Dietary Inflammatory Index and cardiometabolic and inflammatory profiles in children and adolescents with type 1 diabetes: a cross-sectional study
Source: Pediatr Res. 2026 May 8;100(3):1198–206. doi: 10.1038/s41390-026-05069-2 (PMC13672054; doi:10.1038/s41390-026-05069-2)
Supplement: Supplementary file 1 [file 41390_2026_5069_MOESM1_ESM.pdf]

Dietary Inflammatory Index and Cardiometabolic and Inflammatory Profiles in Children and Adolescents With Type 1 Diabetes: A Cross-Sectional Study

Supplementary Material

Table S1

Dietary inflammatory index parameters

	Inflammatory effect score	Global daily mean intake (unit/day)	Standard deviation
Energy (kcal)	0.180	2056	338.0
Protein (g)	0.021	79.4	13.9
Total fat (g)	0.298	71.4	19.4
Saturated fat (g)	0.373	28.6	8.0
Mono unsaturated fat (g)	-0.09	27.0	6.1
Polyunsaturated fat (g)	-0.337	13.8	3.76
n-3 (g)	-0.436	1.06	1.06
n-6 (g)	0.159	10.8	7.50
Cholesterol (mg)	0.110	279.4	51.2
Carbohydrates (g)	0.097	272.2	40.0
Dietary fiber (g)	-0.663	18.8	4.9
Vitamin A (RE)	-0.401	983.5	518.6
Beta-Karoten (µg)	-0.584	3718	1720
Vitamin D (µg)	-0.446	6.26	2.21
Vitamin E (mg)	-0.419	8.73	1.49
Thiamin (mg)	-0.098	1.70	0.66
Riboflavin (mg)	-0.068	1.70	0.79
Niacin (mg)	-0.246	25.9	11.7
Vitamin B6 (mg)	-0.365	1.47	0.74
Folic acid (µg)	-0.190	273	70.7
Vitamin B12 (µg)	0.106	5.15	2.70
Vitamin C (mg)	-0.424	118.2	43.4
Iron (mg)	0.032	13.3	3.71
Magnesium (mg)	-0.484	310.1	139.4
Zinc (mg)	-0.313	9.84	2.19
Selenium (mg)	-0.191	67.0	25.1

Table S2

Correlations between total dietary inflammatory index scores and clinical parameters.

	DII total score	
**Demographics**	**r**	**p**
Age (years)	0.142	0.215
Year of diagnosis (years)	-0.007	0.949
Mother age (years)	-0.042	0.714
Father age (years)	0.068	0.555
Number of individuals in the family	-0.013	0.910
Number of children in the family	-0.197	0.085
Ranking of the patient in the family	-0.232	**0.041**
**Anthropometric measurements**		
Weight (kg)	0.167	0.144
Height (cm)	-0.011	0.926
Body mass index (kg/m^2)	0.420	**0.025**
Waist circumference (cm)	0.122	0.286
Waist-to-height ratio	0.151	0.187
Hip circumference (cm)	0.175	0.125
Triceps skinfold thicknesses (cm)	0.117	0.306
Biceps skinfold thicknesses (cm)	0.114	0.319
Mid-upper arm circumference (cm)	0.200	0.079
Body fat percentage (%)	0.153	0.180
Fat-free body mass (kg)	0.121	0.290
Muscle mass (kg)	0.115	0.321
Total body water percentage (%)	-0.240	**0.034**
**Energy and nutrient intakes**		
Energy (kkal)	0.617	**<0.001**
Water (ml)	-0.111	0.335
Protein (g)	0.424	**<0.001**
Total fat (g)	0.703	**<0.001**
Saturated fat (g)	0.249	0.028
Mono unsaturated fat (g)	0.335	**0.003**
Polyunsaturated fat (g)	0.489	**<0.001**
n-3 (g)	-0.540	**<0.001**
n-6 (g)	-0.329	**0.004**
Cholesterol (mg)	0.753	**<0.001**
Carbohydrates (g)	0.633	**<0.001**
Dietary fiber (g)	-0.609	**<0.001**
Vitamin A (RE)	-0.563	**<0.001**
Beta-Karoten (µg)	-0.528	**<0.001**
Vitamin D (µg)	-0.313	**0.008**
Vitamin E (mg)	-0.563	**<0.001**
Thiamin (mg)	-0.206	0.074
Riboflavin (mg)	-0.437	**<0.001**
Niacin (mg)	-0.521	**<0.001**
Vitamin B6 (mg)	-0.450	**<0.001**

Folic acid (µg)	-0.417	**<0.001**
Vitamin B12 (µg)	0.441	**<0.001**
Vitamin C (mg)	-0.516	**<0.001**
Iron (mg)	0.382	**0.001**
Magnesium (mg)	-0.446	**<0.001**
Zinc (mg)	-0.452	**<0.001**
Selenium (mg)	-0.628	**<0.001**
**Blood parameters**		
Glucose (mg/dL)	-0.103	0.371
HbA1c (mmol)	0.628	**<0.001**
HbA1c (%)	0.629	**<0.001**
HDL (mg/dL)	0.126	0.272
LDL (mg/dL)	0.689	**<0.001**
TG (mg/dL)	-0.089	0.636
Total cholesterol (mg/dL)	0.213	0.061
CRP (mg/dL)	0.121	0,291
Fibrinogen (Mg/Dl)	0.043	0.708
Adiponectin (Ug/Ml)	-0.624	**<0.001**
TNF-α (pg/ml)	0.574	**<0.001**
IL-6 (pg/ml)	0.421	**<0.001**
Leptin (ng/mL)	0.524	**<0.001**

Table S3

Unadjusted biochemical and inflammatory markers by DII tertiles

	T1	T2	T3	p value
Glucose (mg/dL)	232.5 (98.1)	195.9 (98.5)	214.7 (109.6)	0.439
HbA1c (mmol)	57.6 (8.13)	84.4 (12.1)	112.2 (22.2)	**<0.001**
HbA1c (%)	7.42 (0.74)	9.87 (1.11)	12.4 (2.03)	**<0.001**
HDL (mg/dL)	54.2 (16.2)	58.5 (16.55)	56.2 (16.6)	0.646
LDL (mg/dL)	64.7 (5.53)	78.4 (6.87)	100.2 (12.3)	**<0.001**
TG (mg/dL)	103.9 (52.8)	84.8 (37.6)	116.5 (72.4)	0.128
Total cholesterol (mg/dL)	155.3 (28.7)	160.5 (21.2)	169.5 (28.4)	0.151
CRP (mg/dL)	6.46 (3.22)	6.83 (3.47)	7.76 (3.78)	0.390
Fibrinogen (Mg/Dl)	254.8 (34.1)	254.9 (31.7)	255.5 (33.6)	0.997
Adiponectin (Ug/Ml)	2390 (95.1)	2201 (87.0)	2013 (180)	**<0.001**
TNF-α (pg/ml)	194 (83.4)	523 (101)	671 (20.2)	**<0.001**
IL-6 (pg/ml)	1.018 (0.221)	1.577 (0.455)	4.844 (3.745)	**<0.001**
Leptin (ng/mL)	2503 (629)	4264 (627)	5385 (428)	**<0.001**

T1 (-1.55; 1.00), T2 (1.04; 2.03), T3 (2.04; 2.80) are tertiles.
